# Nanoscale interaction of RecG with mobile fork DNA†

**DOI:** 10.1039/c9na00712a

**Published:** 2020-02-11

**Authors:** Zhiqiang Sun, Yaqing Wang, Piero R. Bianco, Yuri L. Lyubchenko

**Affiliations:** Department of Pharmaceutical Sciences, University of Nebraska Medical Center Omaha NE 68198-6025 USA ylyubchenko@unmc.edu; Center for Single Molecule Biophysics, Department of Microbiology and Immunology, University at Buffalo SUNY Buffalo NY 14214 USA pbianco@buffalo.edu

## Abstract

RecG DNA helicase is a guardian of the bacterial genome where it dominates stalled DNA replication fork rescue. The single-stranded DNA binding protein (SSB) is involved in this process and promotes the binding of RecG to stalled replication forks. Atomic force microscopy (AFM) was used to investigate the interaction of RecG and SSB on a mobile fork substrate capable of being regressed. In the absence of proteins, the fork undergoes spontaneous dynamics between two states, defined by the length of the DNA complementarity at the fork. The binding of SSB does not affect these dynamics as it binds to single-stranded regions as expected. In contrast, RecG interacts with the two states quite differently. We demonstrate that RecG has two modes of interaction with fork DNA in the presence of SSB and ATP. In the first mode, RecG translocates over the duplex region and this activity is defined by SSB-mediated remodeling of helicase. In the second mode, RecG utilizes its helicase activity to regress the fork, in an ATP-dependent manner, displacing SSB on the ssDNA. Overall, our results highlight two functions of RecG that can be employed in the regulation of stalled DNA replication fork rescue.

## Abstract 2

In the presence of ATP, RecG regresses the fork and displaces SSB in the process.

## Graphical abstract

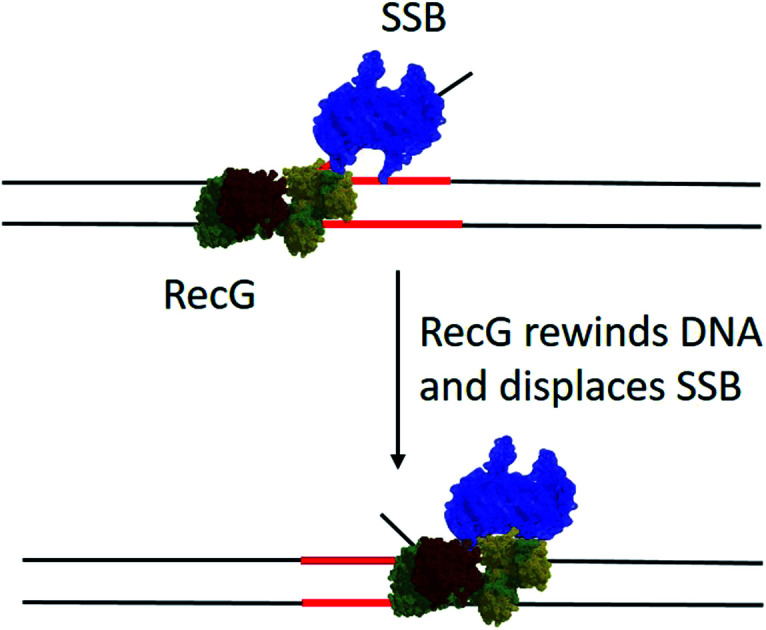

## Introduction

The inherently accurate and highly progressive process of duplication of the genome depends firmly on the cooperation between homologous recombination and DNA repair machinery.^1–3^ The replication machinery can be disrupted due to frequently encountered roadblocks that have the potential to stall or collapse a replication fork. In this case, the importance of interplay arises. Once forks stall, the action of the recombination machinery is needed for fork rescue. RecG catalyzes fork regression which results in the movement of the fork in a net backward direction away from the DNA impediment resulting in the formation of a Holliday junction (HJ). RuvAB binds to the HJ, resulting in additional processing, ultimately resulting in the resurrection of the fork.^4–7^

For regression to occur, RecG forms an intimate complex with the fork.^8^ Here, the wedge domain of helicase binds to the fork in the DNA while the helicase domains are predicted to bind to the parental duplex region ahead of the fork. Modeling of RecG revealed that the enzyme unwinds the replication fork through a structural transition with the helicase domains by hydrolyzing ATP.^9^

In addition to RecG, SSB plays an essential role in fork rescue by enhancing and controlling the activity of RecG in the early stages of the reaction.^6,7,10–12^ We have recently utilized static fork substrates and atomic force microscopy (AFM) to understand how these proteins function at a fork. We demonstrated that the interaction of fork-bound SSB leads to the remodeling of RecG during the loading process onto the DNA. As a result, RecG becomes capable of spontaneous translocation ahead of the replication fork over distances as large as 200 bp and this was directly visualized by time-lapse, high-speed AFM.^13,14^ As the forks used in this study were static, fork regression could not be visualized and in addition, the interplay between SSB and RecG during this process could not be studied.

**Fig. 1:**
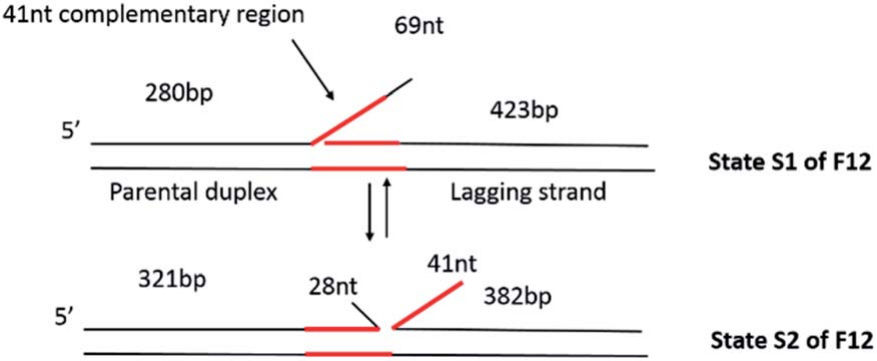
Dynamic fork design to study the fork rescue process. The fork substrate (F12) is comprised of a piece of 3′-end 69 nt ssDNA flanked by duplex regions of different lengths. Within the 69 nt ssDNA, there is a 41 nt region (colored red) complementary to the lagging strand arm of the fork. Consequently, the fork can equilibrate between the two states designated S1 and S2. Interconversion between S1 and S2 involves the formation of duplex DNA between the 41 nt ssDNA and the complement in the lagging strand arm of the fork. This results in a net movement of the fork by 41 bp, from the left to the right in the molecules as shown, concomitant with an increase in the length of the parental duplex and a decrease in the length of the lagging strand arm.

Therefore, to characterize fork transactions under conditions allowing for fork mobility, we constructed a mobile fork substrate containing 41 bp complementarity between the single-stranded tail (leading strand arm of the fork) and the lagging strand which is duplex DNA (Fig. 1). Due to the design of the fork, it was anticipated that it would interconvert between the two states, S1, and S2, driven by spontaneous branch migration. As expected, in the absence of proteins, these two states were directly observed using AFM. Furthermore, in the presence of SSB, a bimodal distribution of the protein position corresponding to the two states of the fork was observed. In the absence of ATP, RecG bound preferentially to one state (state S1), while in the presence of ATP, RecG regressed the fork and displaced SSB in the process. SSB maintains the fork structure (state S2) following regression by RecG. These findings show that DNA helicase couples DNA unwinding to duplex rewinding and also shows the displacement of proteins bound to the DNA, consistent with a previous single-molecule study.^5^

## Methods

### Protein preparation

*RecG* protein was purified as described previously.^7^ Briefly, the protein was eluted using a linear gradient (10–1000 mM NaCl) with RecG eluting between 250 and 360 mM NaCl on a 100 ml Q-Sepharose column equilibrated in buffer A [20 mM Tris–HCl (pH 8.5), 1 mM EDTA, 1 mM DTT, and 10 mM NaCl]. The pooled fractions were then subjected to heparin FF and hydroxylapatite chromatography. Pooled fractions from the hydroxylapatite column were dialyzed overnight in S buffer [10 mM KPO_4_ (pH 6.8), 1 mM DTT, 1 mM EDTA and 100 mM KCl]. The protein was applied to a 1 ml MonoS column and eluted using a linear KCl gradient (100–700 mM) with RecG eluting at 350 mM KCl. The fractions containing RecG were pooled and dialyzed overnight with storage buffer [20 mM Tris–HCl (pH 7.5), 1 mM EDTA, 1 mM DTT, 100 mM NaCl and 50% (v/v) glycerol]. The protein concentration was spectrophotometrically determined using an extinction coefficient of 49 500 M^−1^ cm^−1^.^15^

The SSB protein was purified from the strain K12DH1Dtrp, as described in ref. 16. The concentration of the purified SSB protein was determined at 280 nm using *e* = 30 000 M^−1^ cm^−1^. The site size of the SSB protein determined to be 10 nucleotides per monomer by monitoring the quenching of the intrinsic fluorescence of the SSB that occurred on binding to ssDNA, as described earlier.^17,18^

### Preparation of fork substrate F12

The substrate was assembled from two duplexes and the core fork segment, similar to our previous methodology.^13^ Briefly, the constructs of the two duplexes were precisely the same as the one we used previously.^13,14^ To assemble the core fork segment, four types of ssDNA oligos (O30, O45, O46, and O47) were mixed with the same molar ratio and annealed by heating to 95 °C. The sequences of the oligos are shown in Table 1. The two duplexes and the core fork segment were ligated together in the ratio 1 : 1 : 1 overnight at 16 °C. The final products were purified with HPLC using a TSKgel DNA-STAT column. All oligonucleotides were bought from IDT (Integrated DNA Technologies, Inc. Coralville, Iowa, USA).

**Table 1:** The sequences of different oligos

Oligo name	Sequence
O30	TCATCTGCGTATTGGGCGCTCTTCCGCTTCCTATCT
O45	TCGTTCGGCTGCGGCGAGCGGGATCTAGTAGCTCTGCAGCACTGCATAATTATCAGCTCACTCATA
O46	GCTTATGAGTGAGCTGATAATTATGCAGTGCTGCAGAGCTACTAGATCGCCGCTCGCCGCAGCCGAACGACCTTGCGCAGCGAGTCAGTGAGATAGGAAGCGGAAGAGCGCCCAATACGCAGA
O47	CACTGACTCGCTGCGCAAGGTCGTTCGGCTGCGGCGAGCGGCGATCTAGTAGCTCTGCAGCCTTCATCTTTGGGTTCACTTTCTCCAC

### Preparation of DNA–protein complexes

#### SSB–DNA complexes

DNA (final concentration: 20 nM) was mixed with the SSB tetramer in a molar ratio of 1 : 2, and incubated in 10 μl of binding buffer [10 mM Tris–HCl (pH 7.5), 50 mM NaCl, 5 mM MgCl_2_, and 1 mM DTT] for 10 min.

#### RecG–DNA complexes

DNA (final 20 nM) was mixed with RecG in a molar ratio of 1 : 4, and incubated in 10 μl of binding buffer for 10 min.

#### SSB–RecG–DNA complexes

The SSB tetramer (final 20 nM) and RecG were premixed in the molar ratio 1 : 2 in 30 μl of binding buffer on ice for 30 min. DNA was mixed with the SSB–RecG complexes in a molar ratio of 1 : 2, and incubated in 10 μl of binding buffer for 30 min. The final molar ratio of DNA : SSB : RecG was 1 : 2 : 4.

### AFM imaging and data analysis

#### Imaging

1-(3-Aminopropyl)silatrane (APS) functionalized mica was used as the AFM substrate for all experiments. Briefly, freshly cleaved mica was incubated in 4 ml APS (167 μM) in a cuvette for 30 min and then rinsed with ddH_2_O thoroughly, as described in ref. 14. Ten microliters of the sample were deposited onto APS mica for 2 min, cleaned with ddH_2_O, and dried with a gentle argon gas flow. Images were acquired using tapping mode in air on a MultiMode 8, Nanoscope V system (Bruker, Santa Barbara, CA) using TESPA probes (320 kHz nominal frequency and a 42 N m^−1^ spring constant) from the same vendor.

### Data analysis

The dry sample AFM images were analyzed using the FemtoScan Online software package (Advanced Technologies Center, Moscow, Russia). The positions of SSB were measured from the end of the short arm of the DNA substrate to the center of the protein. The contour lengths of the DNA were then measured from the protein to the other end of DNA. The yield of protein–DNA complexes was calculated from the ratio of compounds to the total number of DNA molecules.

### Sample preparation for time-lapse imaging in liquid with high-speed AFM (HS-AFM)

The freshly cleaved mica was incubated with 2.5 μl of APS for 30 min and then washed with ddH_2_O. The DNA samples (2.5 μl) were then deposited onto APS mica and incubated for 2 min. The sample was then rinsed with 20 μl of the binding buffer. Time-lapse images were acquired using a commercial HS-AFM instrument (RIBM Co. Ltd., Tsukuba, Japan) using custom-built, high-aspect ratio, high-frequency carbon probes (based on BL-AC10DS, Olympus Corp., Tokyo, Japan). The image size was usually set to 300 × 300 nm with 1 nm per pixel resolution, and the scan rate was 800 ms per frame.

## Results

### Experimental design

The fork design used in this study has a 3′-end, 69 nt ssDNA tail inserted between two heterologous duplex regions of different lengths (Fig. 1), similar to the static fork DNA substrate Fork 4 (F4) which we used previously.^13^ We named the new construct Fork 12 (F12). The left duplex region corresponds to parental DNA; the single stranded tail is the leading strand arm, while the right duplex region corresponds to the lagging strand arm of the fork. In contrast to the F4 design, the central core of the F12 substrate is homologous as there is a 41 nt region of ssDNA from the fork position which is complementary to the template lagging strand. This design allows the joint position to move between state S1 and state S2 as shown in Fig. 1. In state S1, the length of the parental duplex (short duplex fragment) is 280 bp, and the lagging duplex length is 423 bp, while that of the ssDNA region is 69 nt. In state S2, the length of the parental duplex (short duplex fragment) is 321 bp and the lagging duplex length is 382 bp, in which case part of the ssDNA (28 nt) anneals to the parental strand and the other part (41 nt) anneals to the lagging strand. This dynamic design is predicted to allow the fork to move between the two states and more importantly, permit the study of the fork regression process.

### The dynamics of F12 between the two states

**Fig. 2:**
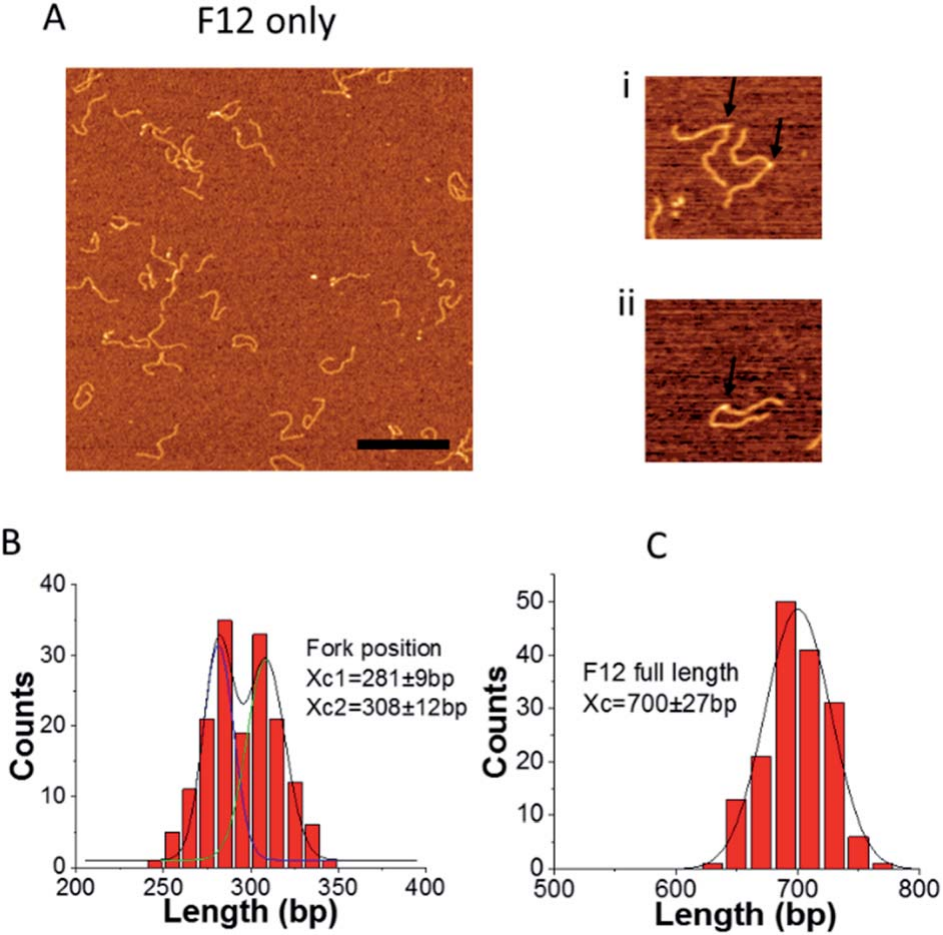
AFM analysis of the fork position for F12 (kinked DNA molecules). (A) Typical AFM images of fork DNA. The bar size is 300 nm. Enlarged images (300 nm × 300 nm) of the selected molecules with a clear appearance of the kinks (indicated with arrows) are shown to the right (i and ii). (B) and (C) are the distributions of fork positions measured from the parental dsDNA end and the full length of F12, respectively. The distributions were fitted by Gaussians and the fitting value *X*_c_, which is defined by the maxima values ± SD, is indicated on the histograms.

To determine whether our design does, in fact, allow fork migration between the two states, we imaged the substrate using AFM. A representative frame of AFM images of F12 on APS mica is shown in Fig. 2. We found some F12 DNA molecules have sharp kinks as shown in the enlarged images (indicated with arrows). The kink can be explained by the nick at the fork joint. The long ssDNA at the fork position can also contribute to the kink formation. This interpretation is supported by the mapping of the kink position by measuring the distance from the end of the parental duplex to the kink position. The distribution of fork positions and the full length of DNA are shown in Fig. 2B and C. The histogram of fork positions can be approximated by using a bimodal Gaussian distribution. The average peak positions are at 281 ± 9 bp and 308 ± 12 bp which correspond to the two states of F12. This result means the fork is dynamic and can move between the two states as designed.

The full length of F12 distribution is shown in Fig. 2C and it is approximated with a single peak Gaussian. The peak position is at 700 ± 27 bp which corresponds to the length of F12 (703 bp). Similar analysis was applied to the immobile fork DNA substrate (F4), which has no complementarity with the ssDNA segment and the data are shown in ESI Fig. S1.† There is only one peak on the fork position distribution with the peak position at 271 ± 16 bp, which is in line with the fork position (280 bp).^13^

### Assembly of a Holliday junction within the F12 template

**Fig. 3:**
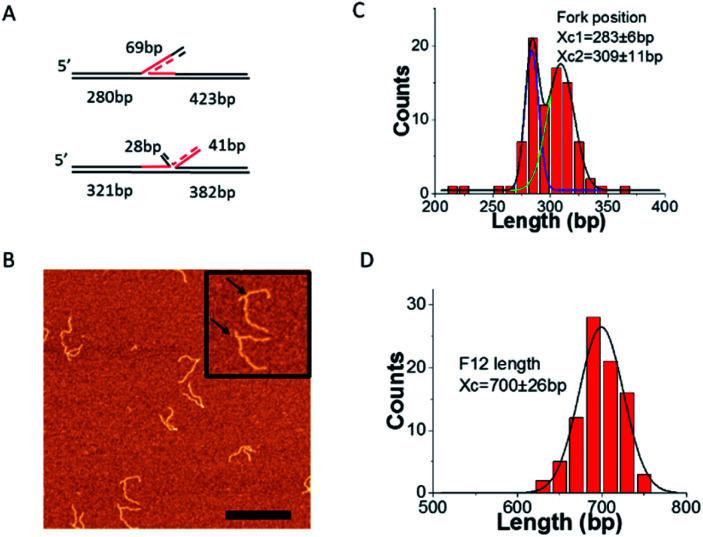
AFM analysis of the fork position on F12 probed by annealing with a complementary 69 nt ssDNA. (A) The scheme of new ssDNA annealed with the two states of F12. (B) AFM images of the annealed fork DNA with 69 nt ssDNA on APS mica. The bar size is 300 nm. Insets show the enlarged images of fork DNA with the annealed double strand fragment (indicated with arrows) at the fork (300 nm × 300 nm). (C) and (D) are distributions of fork positions measured from the end of the short DNA flank and the full length of F12, respectively. Gaussian fits for the length distributions by double peak (C) and a single peak Gaussians (D); the maxima values ± SD are indicated on the histograms.

To further confirm the mobility of the fork position between the two states, a piece of 69 nt ssDNA, which is complementary to the 69 nt ssDNA in F12 was annealed to the fork. Given the self-complementarity to the fork position, the annealing can lead to the structure shown in Fig. 3A. If the fork is in state S1, the new ssDNA will form a three-way junction with F12. However, if the fork is in state S2, a four-way Holliday junction will be formed instead.

The representative AFM images of the annealed DNA are shown in Fig. 3B. The enlarged pictures are the two types of DNA corresponding to different Holliday junctions (indicated with arrows). We performed mapping of the fork position and these data are shown in Fig. 3C. The distribution was fitted with two Gaussians. The average position of the two peaks is at 283 ± 6 bp and 309 ± 11 bp, which are the same as those of the two peaks measured by the kink position on free DNA (Fig. 2B). The distribution of the full length of F12 was fitted with a single peak Gaussian, and the average length is 700 ± 26 bp as shown in Fig. 3D. These data suggest that the fork with the annealed ssDNA complement undergoes dynamic changes between the 3-way and 4-way geometries. This dynamic change of the junction was visualized directly with HS-AFM (ESI movie S1 and selected frames in Fig. S2A†). This dataset demonstrates that the initially highly dynamic three-way junction adopts a four-way junction geometry. In the selected frames in ESI Fig. S2A,† images 1 and 2 correspond to the three-way geometry of the fork, whereas images 3 and 4 show the four-way junction geometry; this conformational transition is illustrated schematically in ESI Fig. S2B.† Additionally, we measured positions of the junctions from the DNA ends (parental DNA strands) and these values are 286 ± 4 bp for three-way junctions (1, 2) and 313 ± 2 bp for four-way junctions (3, 4).

We also annealed the same extra 69 ssDNA to F4 even though the complementary region is only 28 nt on F4. The annealed double strand is still visible as shown in ESI Fig. S3A.† The position was measured and is shown in ESI Fig. S3B.† The average value is still 271 ± 16 bp as expected for the designed fork position.

### The binding of SSB to the F12 fork does not affect fork dynamics

**Fig. 4:**
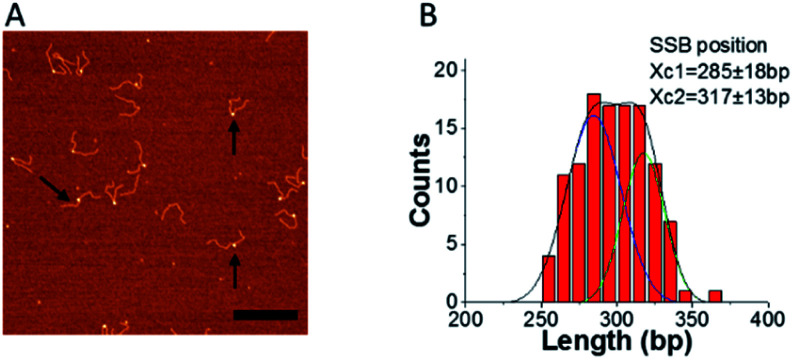
SSB binding does not alter the S1 to S2 equilibrium. (A) Representative image of SSB bound to F12 in the absence of ATP (arrows pointed to some typical complexes of SSB with F12). The bar size is 300 nm. (B) is the distribution of SSB positions obtained from images such as those shown in panel A. The distribution was fitted by double peak Gaussians and the maxima values ± SD are indicated on the histograms.

To ensure that F12 could be used for fork regression analyses, it was first necessary to determine whether SSB affected the equilibrium between states 1 and 2. Therefore, we imaged SSB bound to F12 in the absence of ATP, to mimic fork regression buffer conditions. Typical AFM images of SSB–F12 complexes (indicated with arrows) in the absence of ATP in the binding buffer are shown in Fig. 4A.

SSB appears as a bright feature. The location of the protein on the fork was measured from the end of the parental strand, and the distribution of SSB positions are shown in Fig. 4B. The distribution is broad and can be fitted with two Gaussians. The average SSB positions are at 285 ± 18 bp and 317 ± 13 bp which correlate with the fork position on free DNA (Fig. 1 and 2). This result suggests that SSB binds to the fork in both states and does not affect the migration of the joint fork. The dynamics of SSB on the single F12 was also monitored with time-lapse AFM and displayed in ESI movie S2† (one out of five movies analyzed). The scanning time for each frame is 30 s. The mapping results for SSB on F12 for each frame are showed in ESI Fig. S4A.† In the mapping, the DNA was aligned to the end of the short duplex region. The histogram of the SSB position is shown in the ESI Fig. S4B.† The histogram is well approximated by a single peak Gaussian which suggests that once bound to the DNA, SSB does not slide.

### RecG regresses F12 to state S2 in the presence of ATP

**Fig. 5:**
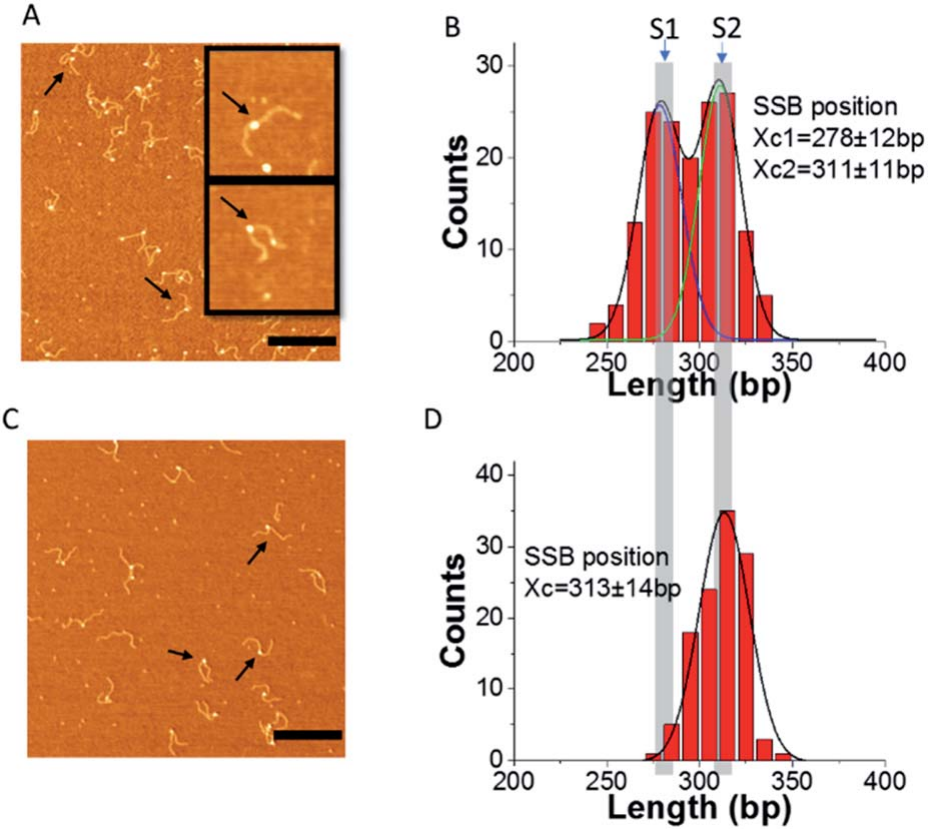
RecG migrates the fork in an ATP hydrolysis-dependent manner. (A) and (C) Representative images of complexes of F12 mixed with SSB and RecG. (A) Absence of ATP; (C) presence of ATP in the binding buffer. The bar size is 300 nm. The two insets in (A) show the typical single and double particles on DNA (300 nm × 300 nm) (arrows pointed to some typical complexes of SSB with F12). (B) and (D) are the distributions of the SSB position obtained from panels (A) and (C), respectively. The histograms are fitted with double and single peak Gaussians and the maxima values ± SD are indicated on the histograms.

Next, we performed experiments in which both SSB and RecG were bound to the same DNA substrate. In these experiments, SSB and RecG were premixed and then F12 was added and the experiments were performed in the absence and presence of ATP. Typical AFM images are shown in Fig. 5A and C.

Most of the DNA–protein complexes appear as particle assemblies along with a few double-particle complexes in which one particle is larger than the other one. The volume of the complexes (single particles and the larger one in double particles indicated with arrows), in Fig. 5A was measured and the distribution is shown in ESI Fig. S5.† The histogram was fitted with a Gaussian, and the average volume is 155.8 ± 30 nm^3^ which is close to the volume of SSB (122.8 ± 22 nm^3^).^14^ This suggests that the single protein or the larger protein in the double particles is SSB, while the smaller “blob” corresponds to RecG, consistent with our previous work.^14^

To check if RecG can change the binding of SSB to F12, we measured the position of SSB on F12, and the histograms are displayed in Fig. 5B and D. In the absence of ATP, the distribution of SSB is still broad and can be fitted with two peak Gaussians. The peak values are at 278 ± 12 bp and 311 ± 11 bp, which are similar to the distribution observed in the complexes of SSB with only DNA. In contrast, when the buffer contains ATP, the distribution of SSB positions is narrow and can be fitted with a single peak Gaussian. The average position is at 313 ± 14 bp which corresponds to state S2 of F12. These results suggest that when RecG is active in the presence of ATP, it can rewind the complementary region on the fork substrate, displacing SSB from the ssDNA.

To investigate if the shift in the position of SSB was due to the regression of DNA by RecG, we performed experiments in the presence of the non-hydrolysable analog ATPγS, instead of ATP. First, the position of SSB alone in the presence of ATPγS was determined and is shown in ESI Fig. S6.† The histogram was fitted with a two-peak Gaussian. The average positions are at 289 ± 7 bp and 313 ± 8 bp, which are very close to the positions of SSB in the absence of ATP. As a control, to determine whether ATP can change the binding activity of SSB to F12, the complex of SSB–F12, in the presence of ATP, was prepared, and SSB positions determined (ESI Fig. S7†). The fitting of the histogram also shows two peaks of SSB positions on F12, and the averages are at 290 ± 16 bp and 316 ± 7 bp. The distribution of the SSB position on F12 is the same in the presence and absence of ATP or ATPγS in the binding buffer. This result suggests that ATP does not affect the position of SSB on F12.

Similar experiments were done for RecG to determine whether the fork position is altered in the presence of nucleoside triphosphate (ESI Fig. S8†). In the absence of ATP, the RecG position on F12 can be fitted with a single peak, and the position at 281 ± 16 bp corresponds to state S1 of F12 (ESI Fig. S8A†). In the presence of ATP, the distribution of RecG is broad and can be fitted with two Gaussians. The peak positions are at 280 ± 15 bp and 310 ± 16 bp. The new maximum at 310 bp suggests that RecG binds to the fork and regresses it from state S1 to state S2. The results suggest that, in the absence of ATP, RecG state S1 mimics an ssDNA gap on the leading strand, consistent with previous work.^5^ In contrast, in the presence of ATP, RecG catalyzes the regression of the fork from state S1 to S2. However, due to the design of the fork, a fraction of the regressed fork reverts back to S1 once RecG disengages from the DNA.

### RecG binding to the F12 fork in the presence of SSB and ATP

**Fig. 6:**
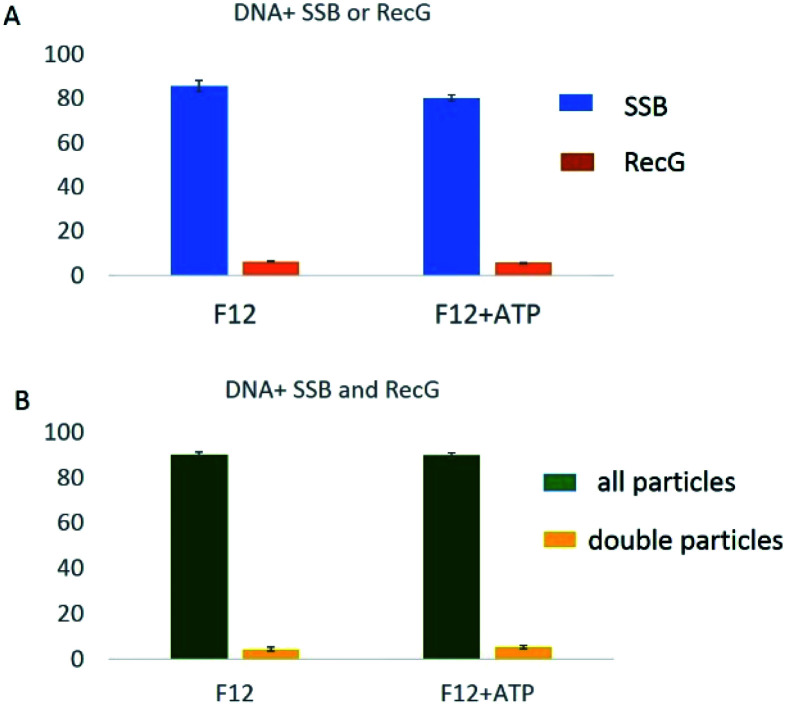
The yield of complexes for different DNA protein mixtures. (A) The columns show the yield of F12 with SSB (blue) or RecG (orange) when DNA was mixed with only one protein without and with ATP in the buffer. (B) The columns show the yield of SSB (all particles) and RecG (double particles) when DNA was mixed with SSB and RecG without and with ATP in the buffer.

To characterize the coupling of RecG catalyzed fork regression and the interaction with SSB, additional analysis was performed. Consequently, the positions of SSB and RecG on the fork in the complexes containing both proteins as well as the yields of each complex were measured. Fig. 6A presents the results of F12 mixed with SSB or RecG only. The yields of SSB–F12 complexes (blue columns) were similar to each other in the absence (left) and the presence (right) of ATP in the buffer. When F12 was mixed with both SSB and RecG, the yield of RecG–F12 complexes was counted by the complexes which contained double particles on the same DNA strand (similar to the methods with F4 (ref. 13 and 14)). The yield of SSB–F12 complexes was obtained by counting all the complexes including single or double particles. The yield of complexes of SSB only with F12 (Fig. 6A) is identical to the overall yield of complexes in the mixture of SSB and RecG together with F12 (Fig. 6B). Similarly, the yield of complexes of RecG only with F12 (Fig. 6A) is identical to the yield of double-particle complexes in the mixture of SSB and RecG together with F12 (Fig. 6B). Note that the presence of SSB did not increase the binding of RecG to the fork substrate which is different from our previous data using a static fork F4 in which the SSB mediated remodeling of RecG was the major factor defining the elevated yield of RecG complexes with DNA.^14^ This finding suggests that the dynamics of the F12 fork between the states S1 and S2 is a factor contributing to the RecG remodeling process.

We also analyzed maps of complexes containing both SSB and RecG (ESI Fig. S9 and S10†) which can be distinguished by the different sizes of the AFM images. The maps show that SSB (larger blobs) and RecG (smaller blobs) localize to different places on F12 DNA. We measured the position of both SSB and RecG on DNA as well as the arm length of F12 on both sides of SSB. The mapping of SSB and RecG on F12 is shown in ESI Fig. S9B and S10B.† In the mapping, the DNA was aligned to the position of SSB. RecG was observed to localize to both sides of SSB suggesting that RecG translocates on F12. The distance between SSB and RecG was measured and the data are shown in ESI Fig. S9C and S10C.† The SSB position was assigned to “0” in these maps. When RecG is on the parental strand, the value of the distance is negative, and when RecG is on the nascent strand, the value is positive. Analysis shows that the histograms of the SSB–RecG distance are similar in the absence and presence of ATP. The RecG position appears broadly on F12 and is expected for an active process on a dynamic fork. As we reported earlier,^13^ RecG translocates to the side of SSB in both directions in an ATP independent manner. This is another difference between the two types of forks, pointing to the role of the dynamics of the fork in the assembly of RecG with the fork.^13^

## Discussion

The primary conclusion of this AFM study is that RecG drives fork regression and in the process, displaces SSB from DNA. The AFM results are consistent with previous single-molecule studies with magnetic tweezers.^5,19^ By using a static fork substrate in the previous study, we demonstrated that SSB facilitates the loading of RecG onto the fork and in the process, remodels helicase.^14^ Remodeling involves binding of the linker domain of SSB to the OB-fold of RecG so that fork binding is precluded.^20–22^ Consequently, RecG, with binding predicted to be mediated by the helicase domains, binds preferentially to the parental duplex region ahead of the fork. Importantly, in this remodeled state, RecG is capable of spontaneous migration along the DNA duplex, and we hypothesized that in this new role RecG maintains DNA duplex integrity.^13^ In fact, mismatches in this region impair RecG binding onto the parental DNA duplex (manuscript in preparation).

As a static fork was used in the previous study, it was not possible to study the fork regression reaction catalyzed by this enzyme. Therefore, a mobile fork design was required, designated F12. This fork substrate alternates between two states: S1, corresponding to a stalled fork and S2, corresponding to a regressed fork. Results show that both states formed with equal probability and that SSB bound to each state without a clear preference (Fig. 2–4(B)). The HS-AFM data (ESI Fig. S2†) directly visualize the transition of F12 fork complexed with a ssDNA complement between states S1 and S2.

Binding of RecG to F12 DNA remains transient, so that complex yield remains as low as 6.3% and is unaffected by the presence of ATP. Furthermore, the presence of ATP does not change the partition of F12 DNA between states S1 and S2 (ESI Fig. S8B†). This follows because any action by RecG would result in the transition from state S1 to S2 due to the ability of helicase to regress the fork. However, a fraction of the regressed fork will revert back to S1, thereby reestablishing the equilibrium. In contrast, when SSB is present in the regression reaction, it binds to the product S2, trapping the fork and producing an increase in the ratio of S2 : S1. The RecG-dependent shift in this ratio was only observed in the presence of ATP since the equilibrium between S1 and S2 was unaltered in the absence of ATP or in the presence of the non-hydrolyzable analog, ATPγS. Control experiments demonstrate that SSB alone does not alter the S1 : S2 ratio and it can maintain the status of the fork state after binding to F12. Therefore, the only way this ratio could be altered is if RecG bound to the SSB–DNA complex and then used ATP binding and hydrolysis to drive fork regression, concomitant with SSB displacement.

Similar to previous studies with the static F4 fork,^14^ we were able to observe SSB mediated remodeling of RecG by direct visualization of the complexes of F12 in which the two proteins were bound. We termed these “two-blob complexes” (Fig. 5 and ESI Fig. S9A†). In these complexes, the SSB position coincides with the location of the fork, whereas RecG binds to DNA far from the fork. Note that both proteins appear on the AFM images as globular features of different sizes with SSB being larger than RecG, allowing the discernment of protein identity as shown previously.^13,14^ We mapped the RecG position using SSB as a marker (ESI Fig. S9B†) and the data have shown that remodeled RecG binds to both DNA duplexes with almost the same affinity. Thus, regardless of whether the fork is static or dynamic, SSB-loading of RecG concomitant with helicase remodeling is observed. This suggests that remodeling is intrinsic to the SSB-mediated loading process.

Although the remodeling of RecG by SSB was observed on the dynamic fork, the yield of double-blob complexes was considerably lower when compared with those on the static fork used previously.^14^ This finding suggests that once loaded onto the parental duplex, RecG slides back to the fork and the wedge domain engages the fork, resulting in regression and displacement of SSB, and this dynamics is coupled to ATP hydrolysis. For this to occur, SSB must slide some short distance on the ssDNA tail and away from the fork to permit the wedge domain access. SSB sliding has been demonstrated.^23^ In contrast, RecG sliding prior to the onset of regression can only occur when the duplex DNA is undamaged. Ultimately this ensures that regression does not occur and other repair enzymes must process DNA first.

## Conclusions

In this study, we demonstrated the interaction of SSB and RecG with mobile fork DNA. We found that the mobile fork migrated simultaneously between two different states. The presence of SSB did not change but stabilized the two states of the fork structure. ATP did not affect the interaction of SSB with fork DNA. RecG in the presence of SSB has two different modes of interaction with fork DNA. In the first mode, RecG was remodeled by SSB and translocated along the duplex region. In the second mode, in the presence of ATP, RecG's helicase was active and regressed the fork in an ATP-dependent manner. The regression of RecG displaced the SSB on the ssDNA at the fork.

## Conflicts of interest

There are no conflicts to declare.

## Supplementary Material

NA-002-C9NA00712A-s001

NA-002-C9NA00712A-s002

NA-002-C9NA00712A-s003
