# Supplementary material for: Nanoscale interaction of RecG with mobile fork DNA
Source: Nanoscale Adv. 2020 Feb 11;2(3):1318–24. doi: 10.1039/c9na00712a (PMC8009289; doi:10.1039/c9na00712a)
Supplement: NA-002-C9NA00712A-s003 [file NA-002-C9NA00712A-s003.pdf]

Electronic Supplementary Material (ESI) for Nanoscale Advances.
This journal is © The Royal Society of Chemistry 2020

Supporting Information

Two modes of RecG interaction with fork DNA

Zhiqiang Sun[1], Yaqing Wang[1], Piero R. Bianco[2], and Yuri L. Lyubchenko[1*]*

[1]Department of Pharmaceutical Sciences, University of Nebraska Medical Center, Omaha, NE 68198-6025, USA

[2] Center for Single Molecule Biophysics and Department of Microbiology and Immunology, University at Buffalo, SUNY, Buffalo, NY 14214, USA

*Corresponding authors

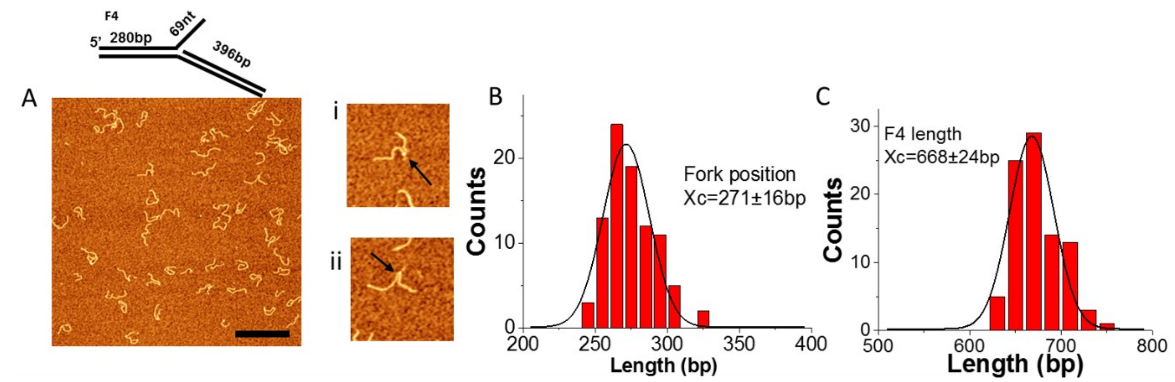

Figure S1. AFM analysis of the fork position on F4 (kinked DNA molecules). (A) shows AFM images of free fork DNA on APS mica (bar size 300nm). Enlarged images (size 300nmx300nm) on the right (i and ii) show F4 with sharp kink at fork joint position (indicated with arrows). (b) shows distribution of fork position measured by the kink position. (C) is the distribution of full length of F4. All the distributions were fitted by Gaussians and the maxima values ± SD are indicated on the histograms.

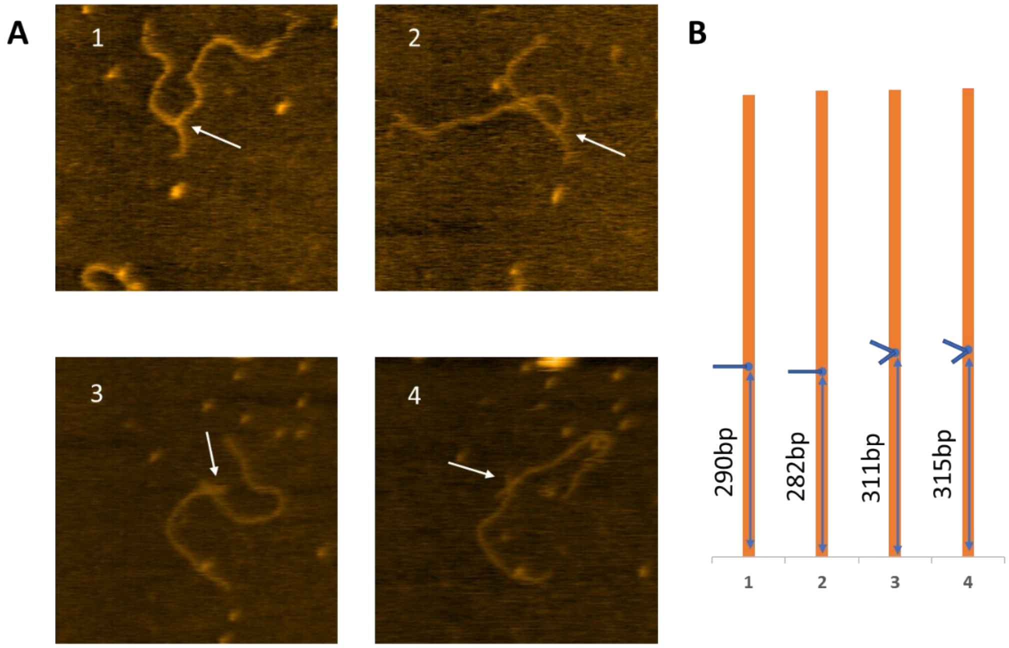

Figure S2. Dynamics of F12 DNA complexed with 69 nt ssDNA complement. (A) Selected HS-AFM images of F12 DNA corresponding to the transition of the junction between the 3-way (1 and 2) and 4-way geometries (3 and 4) (indicated with arrows). (B) The scheme of junction and position change of fork position on DNA was shown. The DNA lengths to the junctions position are indicated in these schemes.

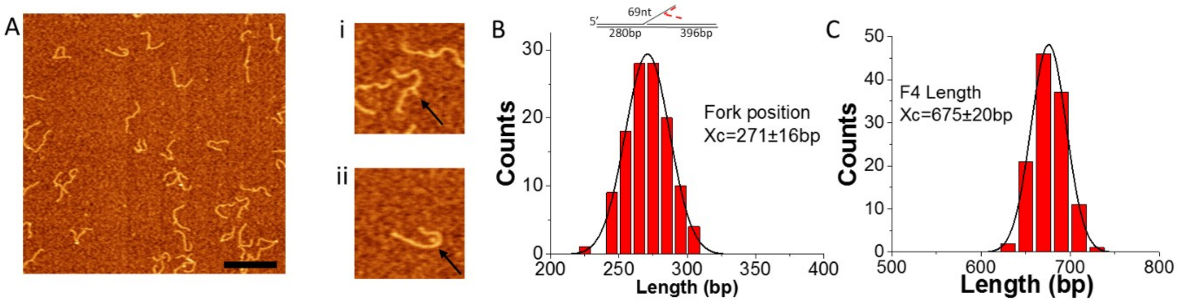

Figure S3. AFM analysis of fork position on F4 complexed with a complementary ssDNA. (A) Typical AFM images of annealed F4 with a ssDNA complementary to the tail DNA on APS mica (bar size 300nm). Zoomed images (size 300nm) on the right (i and ii) show selected images of F4 with annealed duplex region at fork joint position (indicated with arrows). (B) shows distribution of fork position on the annealed duplex DNA. (C) is the distribution for the full length of F4. All the distributions are fitted by Gaussians and the maxima values ± SD are indicated on the histograms.

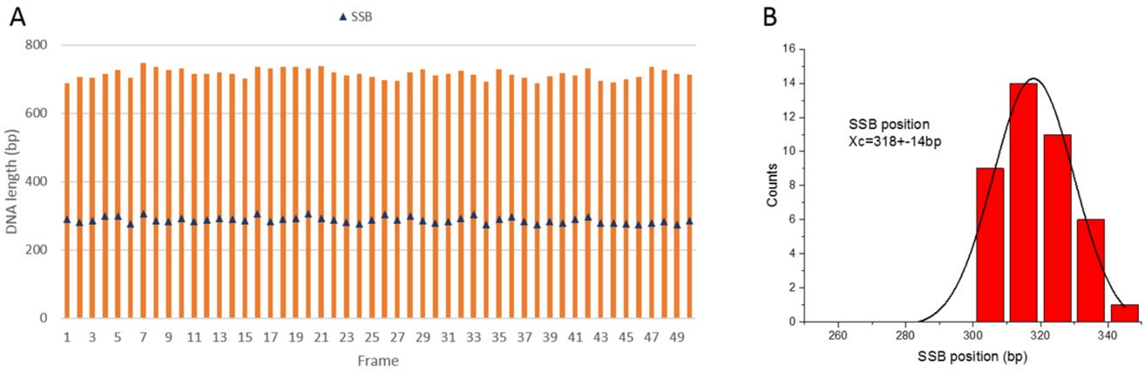

Figure S4. SSB does not change the status of fork state. (A) Mapping of SSB on F12 without presence of ATP in each frame of Supplementary movie 1. DNA was aligned to the end of short duplex arm. The scan time for each frame is 30s. The distribution of SSB relative to the end of short duplex arm distances is shown in (B).

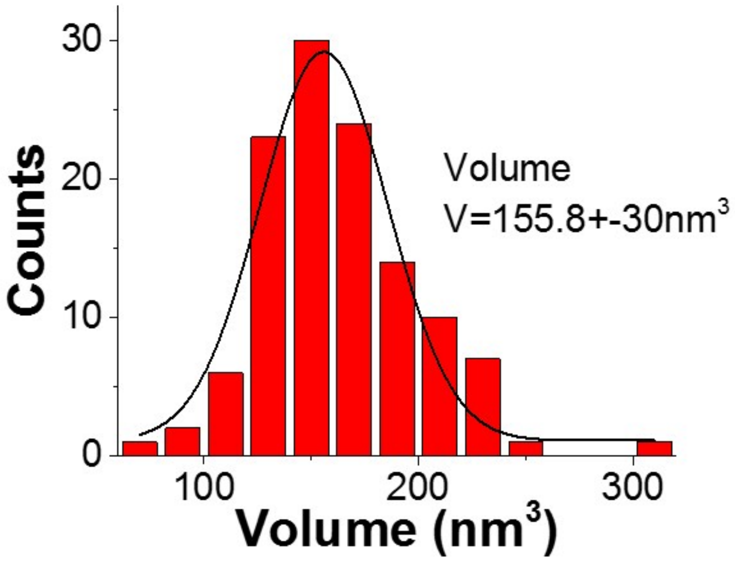

Figure S5. F12 complexes with SSB and RecG in the absence of ATP. (A) shows the distribution of volume of single particles or the bigger particles where there are two particles on same DNA. The histogram is fitted with single peak Gaussians and the maxima values ± SD is indicated on the histograms.

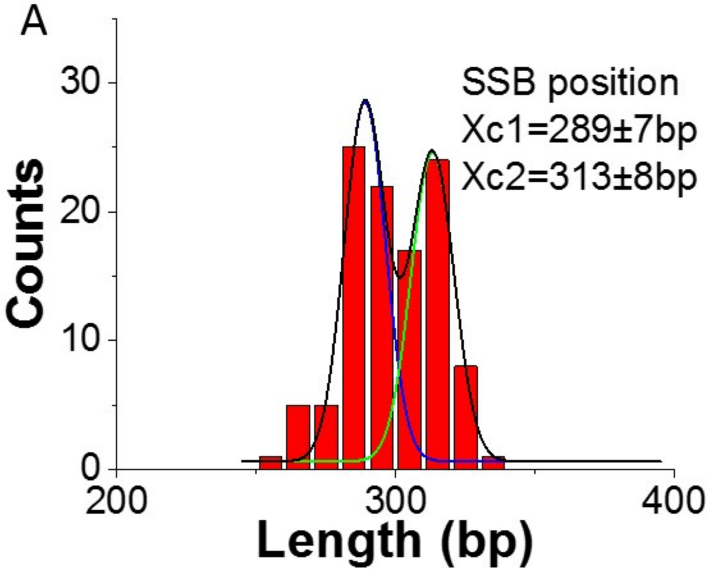

Figure S6. F12 complexes with SSB and RecG in the presence of ATPγS. (A) is the distribution of SSB position on F12. The histogram is fitted with double peak Gaussians and the maxima values ± SD is indicated on the histograms.

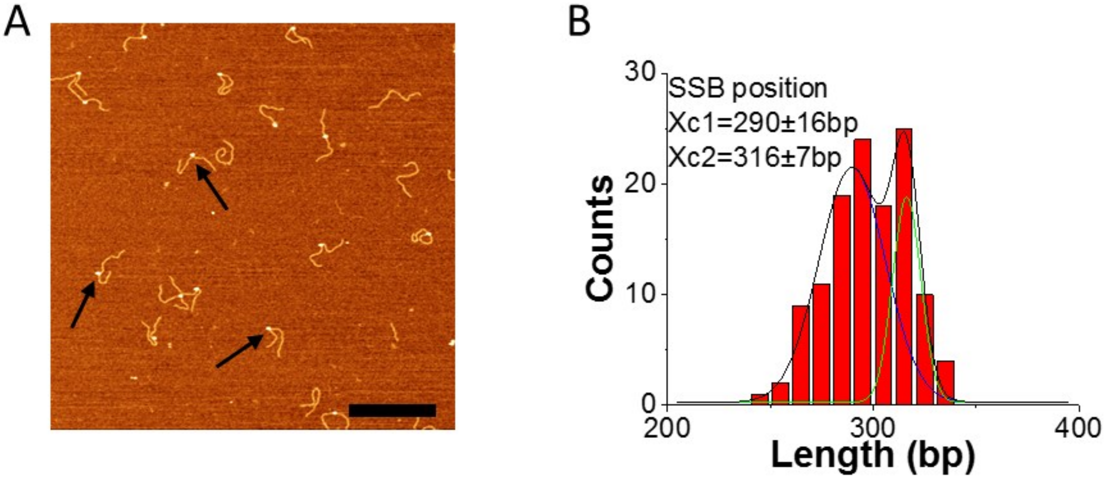

Figure S7. F12 DNA complexes with SSB in presence of ATP. (A) representative the images of SSB with F12 complexes (indicated with arrows) in the binding buffer. Bar size is 300nm. (B) is the distributions of SSB position corresponding to (A). The distribution was fitted by double peak Gaussians and the maxima values ± SD was indicated on the histograms.

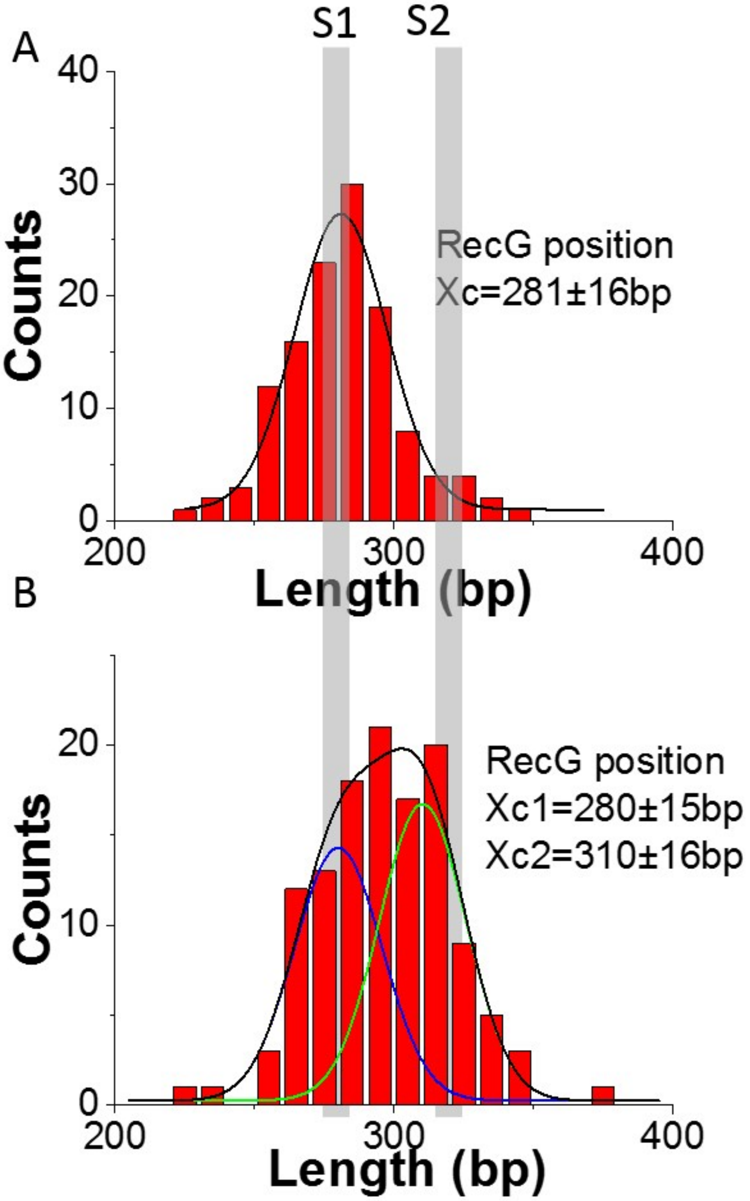

Figure S8. RecG-F12 complexes formed in the absence and presence of ATP. (A) and (B) are the distributions of RecG positions on F12 with no ATP and in the presence of ATP, respectively. The distributions are fitted by single and double peak Gaussians and the maxima values ± SD are indicated on the histograms. The grey shaded bars correspond to the position of two state of F12.

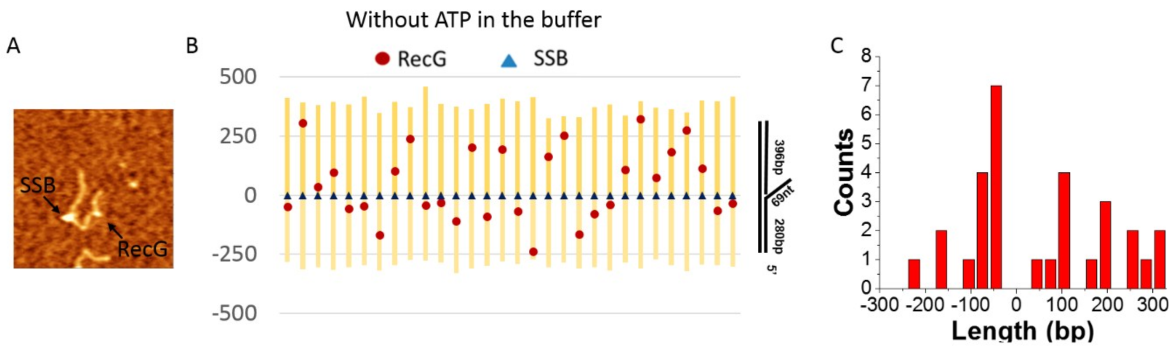

Figure S9. (A) Typical image of double particles on F12 (size 300nm). (B) Mapping of RecG on the double blob complexes for the sample without ATP. RecG position was aligned relative to SSB position. The negative value corresponds to RecG location on the parental strand DNA. The distribution of RecG relative to SSB distances is shown in (C).

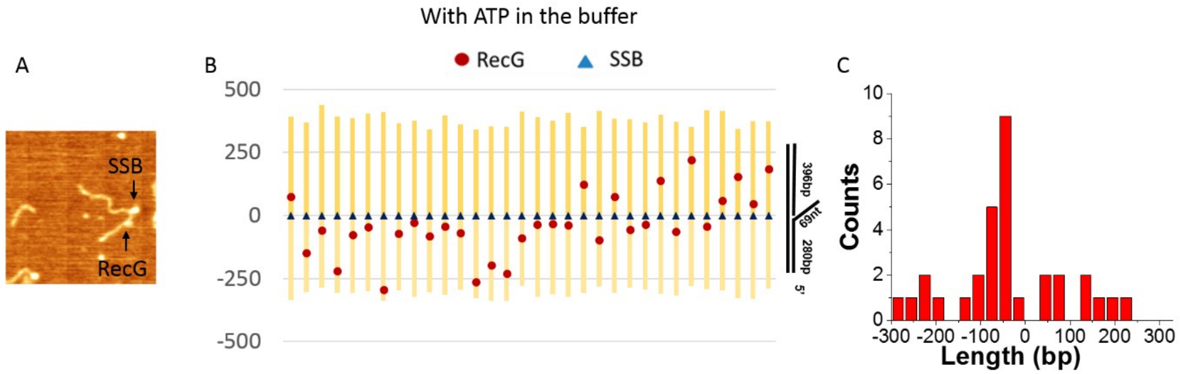

Figure S10. (A) Typical image of double particles on F12 (size 300nm). (B) Mapping of RecG on the double-blob complexes of the sample with ATP in the buffer. RecG position was aligned relative to SSB position. The negative value corresponds to RecG location on the parental strand DNA. The distribution of RecG relative to SSB distances is shown in (C).

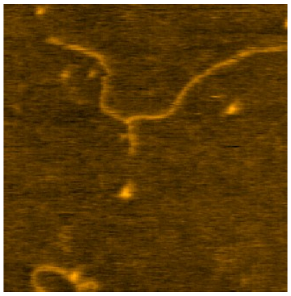

Movie S1. Dynamics of F12 DNA complexed with 69 nt ssDNA complement. The scan time for each frame is 800 ms. The scan size is 200 x 200nm. Selected frames are shown in Figure S2.

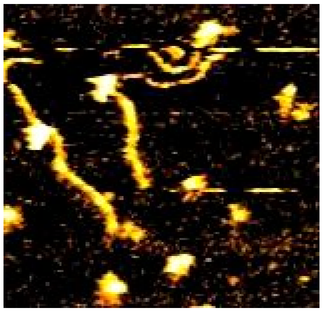

Movie S2. SSB binding does not affect fork dynamics. The movie shows interaction of SSB with F12. SSB binds to the ssDNA at fork position and does not move along F12 duplex. The scan time for each frame is 30s. The scan size is 450nm x 450nm.
